# Physiological State Evaluation in Working Environment Using Expert System and Random Forest Machine Learning Algorithm

**DOI:** 10.3390/healthcare11020220

**Published:** 2023-01-11

**Authors:** Eglė Butkevičiūtė, Liepa Bikulčienė, Aušra Žvironienė

**Affiliations:** 1 Department of Software Engineering, Kaunas University of Technology, 51368 Kaunas, Lithuania; 2 Department of Applied Mathematics, Kaunas University of Technology, 51368 Kaunas, Lithuania

**Keywords:** work ability evaluation, physiological activity, expert system, random forest algorithm, biomedical systems

## Abstract

Healthy lifestyle is one of the most important factors in the prevention of premature deaths, chronic diseases, productivity loss, obesity, and other economic and social aspects. The workplace plays an important role in promoting the physical activity and wellbeing of employees. Previous studies are mostly focused on individual interviews, various questionnaires that are a conceptual information about individual health state and might change according to question formulation, specialist competence, and other aspects. In this paper the work ability was mostly related to the employee’s physiological state, which consists of three separate systems: cardiovascular, muscular, and neural. Each state consists of several exercises or tests that need to be performed one after another. The proposed data transformation uses fuzzy logic and different membership functions with three or five thresholds, according to the analyzed physiological feature. The transformed datasets are then classified into three stages that correspond to good, moderate, and poor health condition using machine learning techniques. A three-part Random Forest method was applied, where each part corresponds to a separate system. The obtained testing accuracies were 93%, 87%, and 73% for cardiovascular, muscular, and neural human body systems, respectively. The results indicate that the proposed work ability evaluation process may become a good tool for the prevention of possible accidents at work, chronic fatigue, or other health problems.

## 1. Introduction

The lack of physical activity is increasing, which diminishes individual and organizational health. Healthy lifestyle is one of the most important factors in the prevention of premature deaths, chronic diseases, productivity loss, obesity, and other economic and social aspects [1]. Common mental disorders such as depression, anxiety, and stress-related disorders have high consequences around the world, which may lead to premature death, cardiovascular diseases, or cancer [2]. In addition, an appropriate working environment plays an important role in recovery, adaptation, and returning to work after various diseases such as COVID-19 [3] or stroke [4], or with disabilities such as hearing loss [5]. The workplace is one of the most important factors to promote physical activity [6]. However, there is a lack of qualitative evidence exploring workability and its connection to nervous, muscular, and cardiovascular systems of the human body.

In the literature, various studies have focused on a particular job or professions. Research such as [7,8] analyze job satisfaction and stress among doctors or dentists. Considering more dangerous industries, such as workers in the construction field, the non-traditional hazards are analyzed as a large impact on health and injuries [9]. These and similar papers describe how working conditions affect health and productivity. Meanwhile, other scientists are more interested in patients with specific pathologies or symptoms. In [10], the outpatients of a hemodialysis center are considered. The program focuses on decreasing dietary sodium and increasing habitual physical activity. Glucose monitoring at the workplace could also be a good tool for exerting positive effects on compliance as well as functioning at work [11]. Other scientific fields are related to elderly people and their work efficiency or retirement plans [12]. These and similar research provide general information and insights. However, most of them use only survey data that may not be applicable to other research groups.

In general, workability can be defined as the individual‘s ability to fulfill the demands of the labor market [13]. The main characteristics that define workability are professional competence, motivation, work requirements, work environment, health, occupational virtues, and attitudes [14]. This research focuses on mental and physical health factors and proposes a new framework in measuring individual work abilities.

The structure of this paper is as follows: Section 2 highlights the recent literature on work ability and physiological activity in workplaces evaluation techniques. Experimental design, together with the data description and proposed methods are presented in Section 3. Section 4 consists of the data analysis and transformation using membership functions, the Random Forest algorithm, and its implementation to the health feature classification process. Finally, the discussion and conclusions for this paper are presented in Section 5.

## 2. Related Work

A World Health Organization Heath and Work Performance Questionnaire (HPQ) was designed as a self-report instrument to estimate the relationship between workplace environment and health problems [15]. The main focus was on reduced job performance, sickness absence, and work-related accidents/injuries. The reported results show that HPQ generates meaningful measures of work performance and absenteeism. Even though the HPQ may be used to detect and monitor the overall effects of allergies, migraine, etc., it cannot tell what aspects of performance are affected (e.g., motor skills, concentration, memory, etc.).

The literature studies related to workability may be divided into two main categories that focus on Mental Health Problems (MHP) and Physical Health (PH) or disabilities. In [16] the study had a qualitative design based on a phenomenographic approach. The study revealed that a structured interview may identify two main categories: experiences of employees with MHP and strategies to handle the effects of MHP in the workplace. However, the study included only 12 participants, which made it hard to draw overall conclusions. Additionally, the interviews could be more time consuming to make them applicable to a higher number of participants. A higher number of participants were included in [17], where work ability perception and cognitive performance were considered. The results showed that work-directed interventions must be applied to work ability perception to improve mental health conditions.

Previous studies also indicated a relationship between corrective exercises and neck/shoulder pain, sick leave, posture, muscular activity, and workplace pain in office workers [18]. The findings revealed significant changes in the eight-week corrective exercise program and confirmed that the supervised intervention could be more effective than the un-supervised intervention. Team sport activities in the workplace may also be a good health promoting method that influences physical activity behavior and cognitive skills [6].

There have been some exploratory studies on electrocardiographic (ECG) and electroencephalographic (EEG) signals and workload analysis to alert to possible human errors and accidents [19]. Another study [20] combined questionnaires (general heath and international physical activity, occupational physical activity), human physical characteristics (such as body mass index, diastolic and systolic blood pressure, waist circumference), television, and other factors to analyze workplace activity. These and similar studies provide strong evidence that the workplace is a prime area where interventions to reduce sitting time and increase physical activity should be introduced.

Various healthcare systems that consist of different sensors, such as electrodes and location-based and motion sensors, always involve imperfection and uncertainty that may cause wrong inference on the environment. The uncertainty needs to be minimized for better representation of the knowledge of the patient. To avoid fluctuation in variable values, the multi-grade fuzzy approach was selected. Fuzzy logic is a good tool when data encompass great complexity and takes the inadequate information into consideration to overcome imprecise inputs [21]. Multi-grade fuzzy logic has been used in different healthcare fields and contexts. For example, in [22] a pilot study of healthcare service quality was analyzed using a questionnaire of twenty-three criteria. Others use fuzzy-logic techniques together with neural networks. For example, [23] predicted the deterioration of reaction state when people have neurological movement disorders and [24] assessed nutrition-related factors to determine the likelihood of geriatric patient health risks associated with specific syndromes.

In this paper, a novel framework is presented for the work ability evaluation process, where fuzzy logic expert system and machine learning techniques are combined. The presented membership functions are designed for the data transformation, where different thresholds depend on health conditions. All transformed feature values are classified using the Random Forest algorithm, and recommendations are proposed. To make sure that all physiologic features are evaluated, the cardiovascular, muscular, and neural systems were analyzed.

## 3. Materials and Methods

In this paper, the proposed technique divides the initial dataset into three main categories$:\;C_{1}$, $C_{2}$, and $C_{3}$, which correspond to a good health state ($C_{3}$), moderate health condition ($C_{2}$), or health condition with drawbacks ($C_{1}$). A novel framework consists of three main steps:

Data transformation. Expert system evaluation using thresholds of each feature (membership functions). The data are converted into scores (from 1 to 5). The final grade of the physiological state for every person is the sum of scores of all features.

Three-class data split. The final grades of each person are divided into three classes corresponding to the first and third quartiles. The $C_{1}$ class contains 25% of the data that is below the middle number between the minimum value and the median (also known as the lower quartile, Q1). The $C_{3}$ class corresponds to the third quartile (also known as the upper quartile, Q3), which is the middle value between the median and the maximum value [25]. The $C_{1}$ and $C_{2}$ classes represent a poor health condition that need to be improved. Meanwhile, $C_{3}$ contains the rest of the data and represents the appropriate health state.

Random Forest optimization. Using the RF model, the main features are extracted and classified according to the initial classes: $C_{1}$, $C_{2}$, and $C_{3}$.

### 3.1. Data Gathering and Statistics

The designed system consists of a testing environment that includes sensors (Polar belt V10), smart device (mobile phone or tablet), and a specialist client device. The data are gathered using Polar belt V10 and a mobile application that could be installed on the smartphone or tablet. When all tests are finished, the data are sent to the cloud-based system where specialists can see the results and statistics of all participants and make additional evaluation if needed. After the evaluation process, each person receives feedback about their health condition and recommendations on how to improve it. All this information is visible in their smart device.

For the period 2019–2021, there were five events where 98 recruited workers were tested: 57 female, 41 male, age 38.02 ± 11.77 years, height 173.94 ± 9.76 cm, weight 75.75 ± 14.72 kg. In addition, they were asked to fill the SF 36, Baecke physical activity and pain questionnaires and indicate the type of work they did. From all workers, 61 percent indicated that they did sports, including walking, running, tennis, yoga, etc. In indication of type of work (scale from 1 to 5), average of sedentary work was 4.41, standing work was 2.59, working with weights was 1.49, and a job where it was necessary to walk was 3.01. Finally, workers indicated 2.64 (scale from 1 to 5) that after the working day they were physically tired.

**Figure 1:**
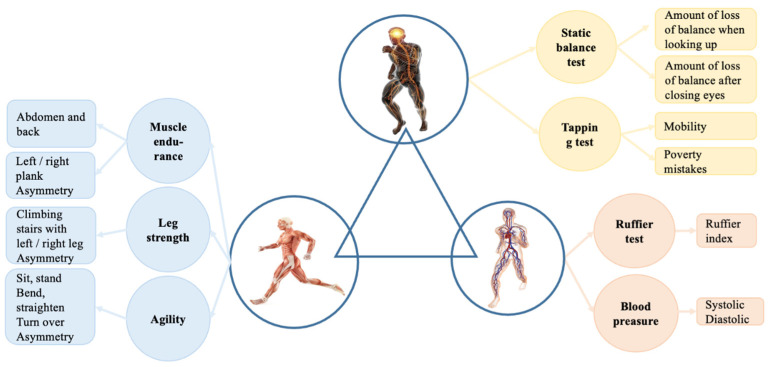
Three-part human body system and exercises that represent each part.

Tests and measurements were divided into three groups according to the complex systems [26,27] approach, where the human body consists of three main parts: cardiovascular, muscular, and nervous systems. By this theory, tests and exercises were split into three parts, as shown in Figure 1. In the muscular system, muscle endurance, leg strength, and agility are evaluated. The nervous system evaluation process consists of static balance and tapping tests. Finally, in the cardiovascular system, the Ruffier test [28] and blood pressure measurements are considered.

The statistics of different parameters are presented in Table 1, where average values and standard deviations are shown. Different features have various metrics that lead to high differences in scale: some values barely reach value 1 (such as static balance test with left or right leg while eyes are open), and others gain high values (like in the back endurance test). To overcome the scaling issue in the health evaluation process, all feature values were transformed using the membership functions of the proposed expert system

### 3.2. Fuzzy Logic

**Figure 2:**
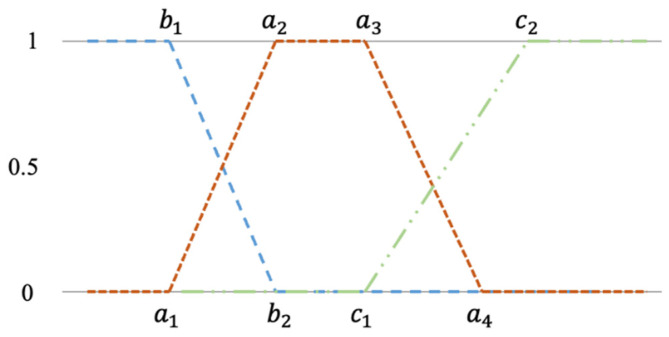
Membership functions and variables.

Fuzzy logic is a class of logic operations where the truth values of variables may be any real number between 0 and 1 [22]. Triangle or trapezium membership functions are convenient for the consensus and group-based decisions regarding fuzzy set definition [21]. In this paper, multi-level trapezium membership functions are defined for both inputs and outputs. The trapezium membership function is used to compute fuzzy membership values of the sensor data (1) and consists of five linear functions:

(1) $$f\left(x;a_{1},a_{2},a_{3},a_{4}\right)=\{\begin{array}{c} \begin{array}{cc} 0, & \;x<a_{1} \end{array}\\ \begin{array}{cc} \frac{x-a_{1}}{a_{2}-a_{1}}, & \;a_{1}\leq x<a_{2} \end{array}\\ \begin{array}{c} \begin{array}{cc} 1, & \;a_{2}\leq x<a_{3} \end{array}\\ \begin{array}{cc} \frac{x-a_{4}}{a_{3}-a_{4}}, & a_{3}\leq x<a_{4} \end{array}\\ \begin{array}{cc} 0, & \;x\geq a_{4} \end{array} \end{array} \end{array};$$

(2) $$f_{left}\left(x;b_{1},\;b_{2}\right)=\{\begin{array}{c} \begin{array}{cc} 1 & \;x<b_{1} \end{array}\\ \begin{array}{cc} \frac{x-b_{2}}{b_{1}-b_{2}} & b_{1}\leq x<b_{2} \end{array};\\ \begin{array}{cc} 0 & \;b_{2}\geq x \end{array} \end{array}$$

(3) $$f_{right}\left(x;c_{1},\;c_{2}\right)=\{\begin{array}{c} \begin{array}{cc} 0 & x<c_{1} \end{array}\\ \begin{array}{cc} \frac{x-c_{2}}{c_{1}-c_{2}} & c_{1}\leq x<c_{2} \end{array};\\ \begin{array}{cc} 1 & c_{2}\geq x \end{array} \end{array}$$

where $a_{1},\;a_{2},\;a_{3},\;a_{4}$ or $b_{1},\;b_{2}$ or $c_{1},\;c_{2}\;$are the membership value for each input, *x*. The side membership functions are represented in (2) and (3) formulas as a left and right curve, respectively. Both curves consist of three linear functions. The graphical representation of all possible membership functions is shown in Figure 2. It should be noted that each feature has both side membership functions (left and right) and one or three trapezium functions.

### 3.3. Machine Learning Approach Using Random Forest Algorithm

In this paper, the proposed framework consists of the data transformation using membership functions. All physiological data were scaled from 1 to 5 according to different thresholds that were defined by the expert system. In the next iteration, the data are divided into three categories that represent good, moderate, or poor health condition. All scores were summed, and the final grade was estimated. Total values that are lower Q1 (lower quartile) or higher than Q3 (upper quartile) are considered as bad or good health conditions with class names $C_{1}$ and $C_{3}$, respectively. Other data that fall between whose two quartiles are marked as class $C_{2}$ and represent a moderate health condition that might be easily improved by following proposed recommendations. Meanwhile, if the score appears in class $C_{1}$, most likely a person has several health issues and needs to contact a doctor. This type of recommendation should also appear in the designed expert system. The formula for the proposed data transformation technique is shown below:

(4) $$f\left(x\right)=\{\begin{array}{c} 0,\;x\in \left[min;\;Q1\right]\\ 1,\;x\in \left[Q1;\;Q3\right]\\ 2,\;x\in \left[Q3;\;max\right] \end{array};$$

where *min* and *max* are the minimum and maximum values of *x*; $Q1=\frac{1}{4}\left(n+1\right)$; $Q3=\frac{3}{4}\left(n+1\right)$, and *n* is the total number of *x* values. It should be noted that value 0 represents values of the $C_{1}$ class, 1 represents $C_{2}$ class values, and 2 indicates the $C_{3}$ class. When data transformation is applied, the machine learning model is constructed for the health evaluation process to classify gathered data.

The Random Forest (RF) algorithm is a machine learning technique that consists of an ensemble of randomized classification or regression trees. It generates many decision trees to improve the performance of the final prediction model [29]. Each decision tree corresponds to a set of limits that are hierarchically organized and randomly applied from a root node [30]. One of the most important advantages of using RF classifiers is that this algorithm can model non-linear relationships, and the model may include numerous random decision trees to improve accuracy.

If only two classes are considered in the classification task, the confusion matrix consists of four categories: True Positive (TP) (refers to the number of predictions where the classifier correctly predicts the positive class), True Negative (TN) (refers to the number of predictions where the classifier correctly predicts the negative class), False Positive (FP) (refers to the number of predictions where the classifier incorrectly predicts the negative class as positive), and False Negative (FN) (refers to the number of predictions where the classifier incorrectly predicts the positive class as negative).

In the multi-class classification problem, there are no positive or negative classes, and all classes together with their labels are considered to be equal. In this case TP, TN, FP, and FN should be found for each individual class [31]. In this paper, the three-class problem is discussed, where the confusion matrix can be described as shown in Table 2, where $TC_{1}$, $TC_{2}$, and $TC_{3}$ refer to the number of predictions where the classifier correctly predicts classes $C_{1}$, $C_{2}$, and $C_{2}$ respectively. In addition, $FC_{i}C_{j}$, where i≠j and i,j=1,2,3, corresponds to the faulty predicted *i*-th class as the class *j*.

Using metrics that are presented in Table 2, for each class *i*, the true positive rate ($TP_{i}$), the false positive rate ($FP_{i}$), and the false negative rate ($FN_{i}$) are defined as follows:

(5) $$TP_{i}=TC_{i},$$

(6) $$FP_{i}=\overset{r}{\underset{\begin{array}{c} j=1\\ j\neq i \end{array}}{\sum }}FC_{i}C_{j},$$

(7) $$FN_{i}=\overset{r}{\underset{\begin{array}{c} j=1\\ j\neq i \end{array}}{\sum }}FC_{j}C_{i},$$

where r is the number of classes. In the multi-class classification task, the precision and recall values are averaged [32]. The micro-averaged precision (mi P) and micro-averaged recall (mi R) values are defined below:

(8) $$mi\;P=\frac{\sum_{i=1}^{r}TP_{i}}{\sum_{i=1}^{r}\left(TP_{i}+FP_{i}\right)}=\overset{r}{\underset{i=1}{\sum }}TC_{i};$$

(9) $$mi\;R=\frac{\sum_{i=1}^{r}TP_{i}}{\sum_{i=1}^{r}\left(TP_{i}+FN_{i}\right)}=\overset{r}{\underset{i=1}{\sum }}TC_{i}.$$

Furthermore, the micro-averaged $F_{1}$ score ($mi\;F_{1}$) is defined as the harmonic mean of these quantities:

(10) $$mi\;F_{1}=2\frac{mi\;P\times mi\;R}{mi\;P+mi\;R}=\overset{r}{\underset{i=1}{\sum }}TC_{i}.$$

It should be noted that mi P, mi R, and $mi\;F_{1}$ are all equal to the sum of the diagonal elements of the confusion matrix. Because of this, the micro scale metrics are not very informative. In [33], the alternative multi-class precision and recall definition is proposed. Macro-averaged precision ma P and macro-averaged recall (ma R) are defined as follows:

(11) $$ma\;P=\frac{1}{r}\overset{r}{\underset{i=1}{\sum }}\frac{TP_{i}}{TP_{i}+FP_{i}},$$

(12) $$ma\;R=\frac{1}{r}\overset{r}{\underset{i=1}{\sum }}\frac{TP_{i}}{TP_{i}+FN_{i}}.$$

Finally, the macro-$F_{1}$ score is defined as the harmonic mean of ma P and ma R quantities:

(13) $$ma\;F_{1}=2\frac{ma\;P\times ma\;R}{ma\;P+ma\;R}.$$

Overall accuracy in the multi-class classification problem can be defined as follows [31]:

(14) $$ACC=\frac{\sum_{i=1}^{r}TP_{i}}{\sum_{i=1}^{r}TP_{i}+\sum_{i=1}^{r}FP_{i}+\sum_{i=1}^{r}FN_{i}}.$$

## 4. Results

### 4.1. Proposed Fuzzy System

**Figure 3:**
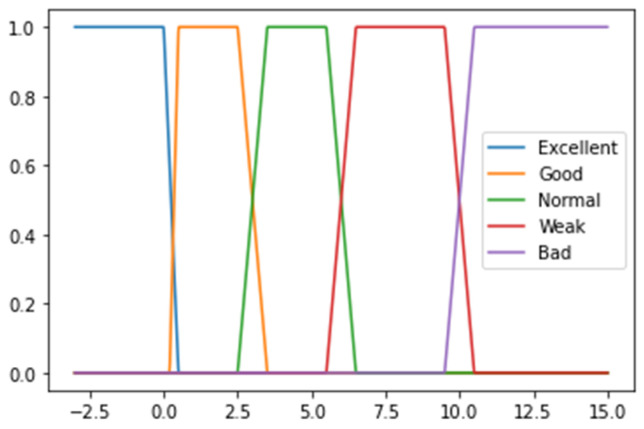
Membership functions using 5 thresholds for the Ruffier index (RFTest) evaluation.

An expert system is part of a computer program that allows the solving of a particular problem by using the knowledge of experts in a specific domain and computational decision procedures. In this article, the described expert system for functional state evaluation is split into three components. In the first part, variables that correspond to cardiovascular system decision rules are described with three input groups: blood pressure and Ruffier index. Parameters for the mental fatigue evaluation expert system values are presented in Table 3. The second component contains parameters of muscular system evaluation. It consists of thirteen input groups. Parameter values for the muscular system evaluation in the expert system are presented in Table 4. The third component contains parameters of the nervous system. It consists of five input groups. Parameter values for the nervous system evaluation in the expert system are presented in Table 5. All fuzzy logic parts are in the same expert decision logic base, depending on possible values (3 or 5), which is composed of logical rules synthesized from the expertise of professionals in work medicine, sports medicine, and rehabilitation. The expert system was prepared based on recommendation from researchers of Lithuanian Sports University and Lithuanian Health Sciences. The components of different systems apply different membership functions with three or five indicators, which are the outcome of previous research and can be expressed as transparent and human-readable logic. The decision output is produced by using a first order weighted average inference engine, which is experimentally validated in Python programming language. In some cases, such as systolic and diastolic blood pressure (see Table 3), the upper and lower values gain a lower score than the middle ones due possible health issues when blood pressure is too high or too low. These threshold values are used to construct the membership functions (see an example in Figure 3).

**Figure 4:**
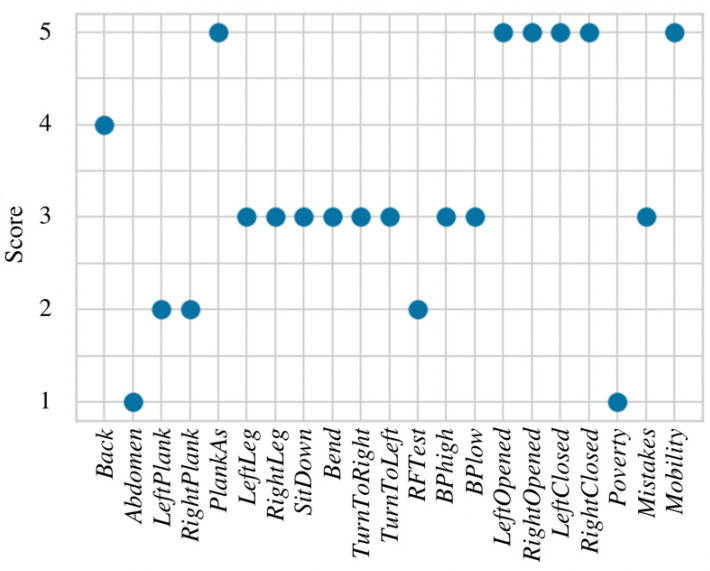
An example of fuzzy logic data transformation.

For the decision-making algorithm, the data are split into categories using five thresholds. Each threshold refers to health evaluation by giving a corresponding score. If the feature value falls into a low value threshold, the score of this part is 1. Low-average value, average value, and high-average value thresholds gain scores of 2, 3, and 4, respectively. Finally, values that fall in the high value threshold are worth 5 points and indicate the perfect value of each feature. Due to the possible data variability, values that are close to the threshold are defined using linear curves with positive or negative slopes (see an example in Figure 3). Gaps between thresholds are not defined in advance and depend on the standard deviation of the feature in each threshold. For example, if the threshold value is 25 and data with values above the threshold have standard deviation equal to 8, then a curve with a negative slope will be drawn between values 21 and 29. An example of data transformation using membership functions is shown in Figure 4, where the total score (sum of all transformed values) is 69.

### 4.2. Expert System Optimization Using Random Forest Method

**Figure 5:**
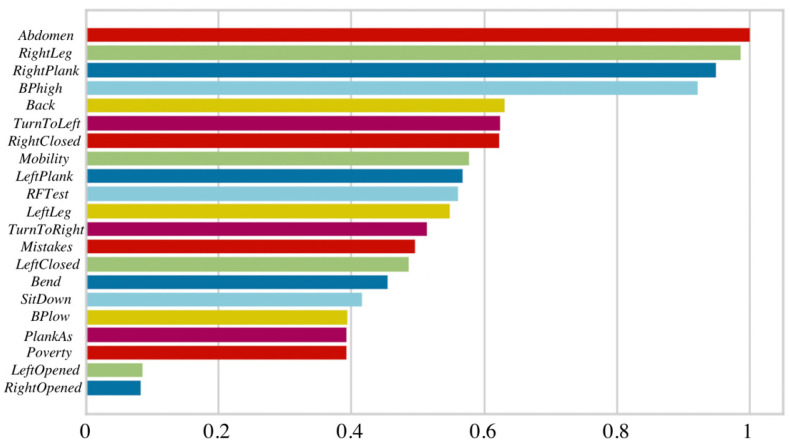
Feature importance for the RF model.

At the beginning of this research, the Random Forest model was constructed and applied to all 21 features at once. The importance of each feature was estimated using entropy [34], where values below 0.05 are considered as not statistically significant and could be removed from the model. According to the obtained results, only variables that belong to the muscular system are considered as important in the RF model (see Figure 5) and cardiovascular, together with neural systems, are not included. This may lead to incorrect health evaluation due to lack of features from other human body systems that are essential in the work ability evaluation process.

**Figure 6:**
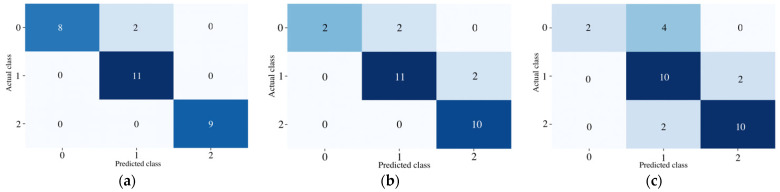
Confusion matrixes for separate systems: (**a**) cardiovascular; (**b**) muscular; (**c**) neural.

In 50 iterations, the average values of overall accuracy, micro and macro precision, recall, and F-1 scores were estimated. The data were split into training and testing datasets by 70% and 30%, respectively. To make sure that the model did not overfit the training data, 5-fold cross-validation was applied to all of the RF models presented in this paper. The results from Table 6 show that the testing accuracies of RF models may vary from 60% to 67% if all features are considered. This led to the conclusion that three systems (cardiovascular, muscular, and nervous) should be evaluated separately, and three new RF models were constructed. Due to the data split, model testing accuracies increased (see Table 6), and the RF model of cardiovascular system even reached 93%. However, health issues of nervous systems are more difficult to detect (reaching only 73% testing accuracy). This might be caused by the low number of features that represent this system or imprecise data split into good, moderate, and poor health conditions. According to these results, three separate models appear to be a better work ability evaluation technique. Examples of testing data classification results into three classes are presented in Figure 6, where the confusion matrices of each system are shown. Furthermore, it can be noticed that muscular and neural systems have a relatively low number of values in class $C_{1}$, which may also result in bad classification accuracies of those models.

**Figure 7:**
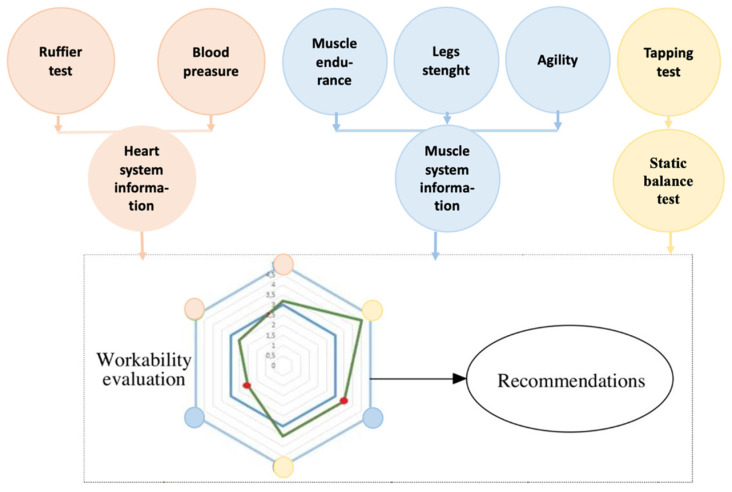
Schematic diagram of the proposed expert system.

The proposed work ability evaluation systems were constructed from separate RF health evaluation models. The final recommendations were generated according to classification results of cardiovascular, muscular, and neural systems. The schematic block of the proposed expert system is shown in Figure 7. The red circles represent the cardiovascular system, blue represents the muscular system, and yellow the neural system. All summed feature values were scaled and put into the hexagon web. Red dots indicate the asymmetry between left and right sides in muscle endurance side plank, static balance, or legs strength tests.

### 4.3. Designed Mobile App Application and Example of Recommendations

**Figure 8:**
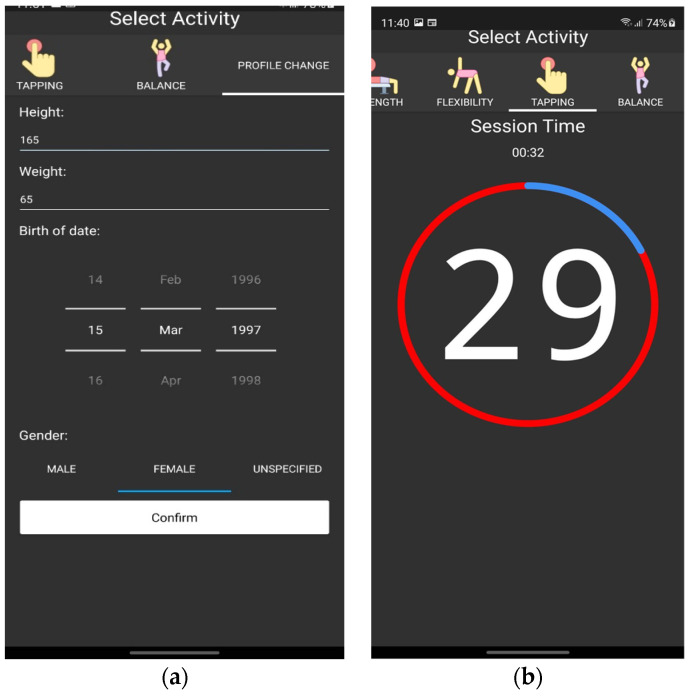
Mobile app: (**a**) registration form; (**b**) an example of evaluation process.

**Figure 9:**
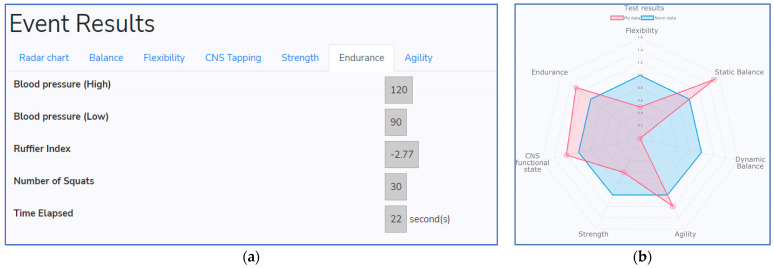
An example of the specialist client device results: (**a**) statistics of the event; (**b**) evaluation results presented as a hexagon web.

The created mobile application consists of the registration form and activity tests (examples are shown in Figure 8). This application has a user-friendly interface and can be installed on a smartphone or tablet. All exercises are listed at the top of the program and are performed one after another. The time and score of every session are visible for the user. After profile information is filled out and all exercises are completed, the data are sent to the specialist client device and all the results, together with basic statistics, are visible for the healthcare specialist (examples are visible in Figure 9). Visual representation is shown in the hexagon web, where blue color corresponds to normal health condition and red color depicts individual health evaluation results (see Figure 9b). The same web is visible for the user in the mobile application.

**Figure 10:**
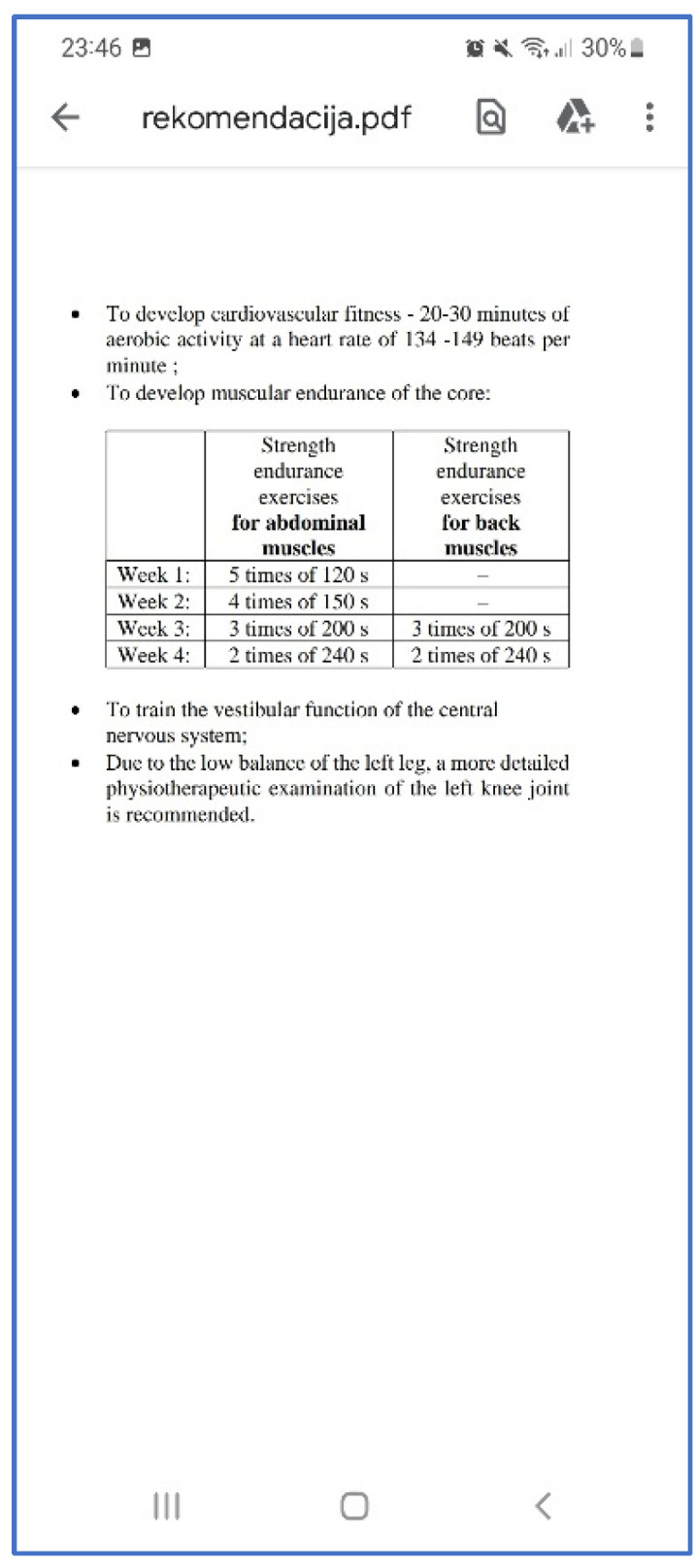
An example of recommendations shown in the mobile application.

An example of listed recommendations to improve work ability is presented in Figure 10. This information is provided in a Portable Document Folder (PDF) format in the client and specialist devices. The training plan is prepared for the 4 week period, and the same tests should be repeated in that time. Furthermore, if there are possible serious health indications, the program suggests contacting the doctor for further investigation to avoid accidents in the workplace.

## 5. Discussion

Previous studies have indicated what affects work ability in general. The research in Finland revealed that employees at ages 30–64 years are mostly affected by health and work environment-related causes [35,36]. Thirty-three percent of work ability is affected by physiological requirements, mental tension, and support. It was noticed that to improve work ability, employers should focus on employees’ health, and interventions should occur even at younger ages [37]. Other studies indicated that common health behavior in recent years was influenced by COVID-19 due to lockdown and social distancing, which caused changes in physical activity [38]. According to these and similar research it could be noticed that physical and mental health are the most important factors in work ability evaluation systems. The same conclusions were obtained in [39], where suggestions were proposed considering that physical exercises via online channels could help to maintain people’s physical activity while they either had to or preferred to stay at home. However, previous studies are mostly focused on individual interviews, various questionnaires that contained conceptual information about individual health state and might change according to question formulation, specialist competence, and other aspects [6,15,16,17,18]. In this paper, the work ability was mostly related to the employee’s physiological state, which consists of three separate systems: cardiovascular, muscular, and neural. Each state consists of several exercises or tests that need to be performed one after another.

In the literature, work ability is mainly related to muscular, cardiovascular, or mental disorders [40]. Even though the human body consists of many other systems, such as the urinary system, endocrine system, etc., studies related to work ability evaluation mainly focus on muscular, cardiovascular, or mental systems. An investigation of the muscular system is often performed to evaluate how disorders might negatively affect abilities to perform daily activities, self-care, and work [41]. Previous research has also shown how stress may seriously affect the autonomic nervous system as well as impacting cognitive performance [42]. The causes of stress might be irregular or long working hour shifts, which are often associated with physiological sleep rhythms alterations [43]. Finally, cardiovascular diseases are commonly related to long working hours and overworking [44]. Even though human working abilities might depend on various factors, this paper also focuses on muscular, cardiovascular, and neural human body systems because they can be easily evaluated with no invasive procedures, additional devices, or long-lasting experiments. The obtained results indicate that the proposed work ability evaluation process may become a good tool for the prevention of possible accidents at work, chronic fatigue, or other health problems.

## 6. Conclusions

The data of this study were gathered from five separate events in Lithuania from 2019 to 2021, where 98 workers (57 female, 41 male, age 38.02 ± 11.77 years, high 173.94 ± 9.76 cm, weight 75.75 ± 14.72 kg) were tested and evaluated. The Baecke physical activity and pain questionnaires were given to all participants, and 61% indicated that they did sports, including walking, running, tennis, yoga, etc. In addition, workers indicated 2.64 (scale from 1 to 5) that after a working day they were physically tired.

In the proposed physiological activity evaluation process, 21 features were selected and analyzed. The realized data transformation technique uses fuzzy logic and different membership functions with three or five thresholds, according to the analyzed physiological feature. The transformed datasets were then classified into three stages that corresponded to good, moderate, and poor health condition using machine learning techniques. Each part was considered as a separate cardiovascular, muscular, or neural system where features were classified using Random Forest algorithms.

It was noticed that the physiological state evaluation process does not necessarily require a huge number of exercises or tests to make sure that necessary recommendations are provided. In the proposed expert system for the cardiovascular system classifier, only three features were included, and 93% accuracy was reached. The results indicate that the proposed work ability evaluation process may become a good tool for the prevention of possible accidents at work, chronic fatigue, or other health problems. Furthermore, the proposed classifiers may help to create a semi-automated recommendation for employees and reduce workload for the medical specialists.

## 7. Limitations

Even though 98 workers were recruited in this study, the number of participants should be expanded to reach higher classification accuracies. In addition, in some studies [38,39] gender, age, and type of work (physical or mental) differences were considered, and statistically significant differences were obtained. Further analysis should also consider these factors, and separate data subsets could be compared.

Furthermore, the proposed framework consists of data transformation and data split into three categories (classes $C_{1}$, $C_{2}$, and $C_{3}$) that are based on the first and third quartiles. However, these metrics may not be the most efficient ones, especially when the data are not distributed equally. Other metrics and data split proportions should be considered in future research.

Finally, the RF model constructed for the neural system is not accurate enough, and only 73% testing accuracy was reached. According to these results, the expert system should be modified by including additional exercises or replacing existing ones. However, this requires further investigation and higher datasets. It is also worth mentioning that working abilities might be affected by other factors that are not evaluated in this research because these do not belong to cardiovascular, muscular, or neural human body systems.

## Figures and Tables

**Table 1:** Basic statistics of each feature in health evaluation system.

Parameter	Mean	Parameter	Mean	Parameter	Mean	Parameter	Mean
Systolic BP, mmHg	124.40 ± 12.99	Left Plank, s	63.06 ± 33.02	Stand up—bend	6.02 ± 0.77	Left eyes closed	2.99 ± 2.58
Diastolic BP, mmHg	77.56 ± 8.33	Right plank, s	65.04 ± 32.87	Turn over (right)	3.57 ± 1.17	Right eyes close	3.08 ± 2.59
Ruffier index	8.88 ± 4.79	Left leg, norm %	19.60 ± 4.03	Turn over (left)	3.51 ± 1.03	Poverty, %	6.52 ± 2.43
Back endurance, s	251.89 ± 64.72	Right leg, norm %	20.45 ± 4.57	Left eyes opened	0.95 ± 0.58	Mistakes, %	3.57 ± 1.28
Abdomen endurance, s	191.96 ± 92.34	Sit down- stand up, s	6.09 ± 0.92	Right eyes opened	0.89 ± 0.43	Mobility, %	6.37 ± 0.74

**Table 2:** Three-class confusion matrix.

		Predicted Class		
		$\mathrm{Class}\;1\;(C_{1})$	$\mathrm{Class}\;2\;(C_{2})$	$\mathrm{Class}\;3\;(C_{3})$
Actual class	Class 1 ($C_{1})$	$TC_{1}$	$FC_{2}C_{1}$	$FC_{3}C_{1}$
	Class 2 ($C_{2})$	$FC_{1}C_{2}$	$TC_{2}$	$FC_{3}C_{2}$
	Class 3 ($C_{3})$	$FC_{1}C_{3}$	$FC_{2}C_{3}$	$TC_{3}$

**Table 3:** Measured parameters and values for the cardiovascular system evaluation in the proposed expert system.

Input Variable (Name)	Resulting Value and Units	Low Value Threshold	Low-Average value Threshold	Average Value Threshold	High-Average Value Threshold	High Value Threshold
Systolic blood pressure (BPhigh)	ABP (mmHg)	<112		>130		112–130
Diastolic blood pressure (BPlow)	ABP (mmHg)	<77		>87		77–87
Ruffier index (RFTest)	Number	>10	6–10	3–6	0–3	<0

**Table 4:** Measured parameters and values for the muscular system evaluation in the proposed expert system.

Input Variable (Name)	Resulting Value and Units	Low Value Threshold	Low-Average Value Threshold	Average Value Threshold	High-Average Value Threshold	High Value Threshold
Back/Abdomen muscles endurance (Back and Abdomen)	s	<120	120–180	180–240	240–300	>300
Left/Right side plank (LeftPlank and RightPlank)	s	<30	30–60	60–90	90–120	>120
Asymmetry of side muscles (PlankAs)	s	>90		50–90		<50
Left/right leg strength (LeftLeg and RightLeg)	% of height	<15		15–20		>20
Sit down-stand up /bend (SitDown and Bend)	s (5 movements)	>10	8–10	6–8	4–6	<4
Turn over right/left (TurnToRight and TurnToLeft)	s	>8	6–8	4–6	3–4	<3

**Table 5:** Measured parameters and values for the nervous system evaluation in the proposed expert system.

Input Variable (Name)	Resulting Value and Units	Low Value Threshold	Low-Average Value Threshold	Average Value Threshold	High-Average Value Threshold	High Value Threshold
Static balance 30 s open/closed eyes/left/right leg standing (LeftOpened, RightOpened, LeftClosed, RightClosed)	Number of mistakes	>11	6–10	3–5	1–2	0
CNS mobility (Mobility)	Max average times/5 first s	<6		6–7.5		>7.5
CNS poverty (Poverty)	Absolute value, %	<5		5–25		>25
Mistakes (Mistakes)	%	>5		3–5		<3

**Table 6:** Averaged RF model accuracy, precision, recall, and $F_{1}$ scores.

Measurement	All Features	Separate Systems		
		Cardiovascular	Muscular	Nervous
Accuracy, ACC	0.63	0.93	0.87	0.73
Micro precision, mi P	0.63	0.93	0.87	0.73
Micro recall, mi R	0.63	0.93	0.87	0.73
$\mathrm{Micro}\;F-1\;\mathrm{score},\;mi\;F_{1}$	0.63	0.93	0.87	0.73
Macro precision, ma P	0.67	0.95	0.89	0.82
Macro recall, ma R	0.60	0.93	0.85	0.67
$\mathrm{Macro}\;F-1\;\mathrm{score},\;ma\;F_{1}$	0.62	0.94	0.86	0.68

## Data Availability

Not applicable.

## Abbreviations

**Abbreviation**	**Definition**
ACC	Overall accuracy in the multi-class classification problem
BP	Blood pressure
CNS	Central nervous system
ECG	Electrocardiogram
EEG	Electroencephalogram
FN	False Negative
FP	False Positive
HPQ	Work Performance Questionnaire
$ma\;F_{1}$	macro-$F_{1}$ score
ma P	Macro-averaged precision
ma R	Macro-averaged recall
MHP	Mental Health Problems
$mi\;F_{1}$	Micro-averaged $F_{1}$ score
mi P	Micro-averaged precision
mi R	Micro-averaged recall
PH	Physical Health
Q1	Lower quartile
Q3	Upper quartile
RF	Random Forest
TN	True Negative
TP	True Positive
